# Pre-analytical issues in the haemostasis laboratory: guidance for the clinical laboratories

**DOI:** 10.1186/s12959-016-0123-z

**Published:** 2016-12-12

**Authors:** A. Magnette, M. Chatelain, B. Chatelain, H. Ten Cate, F. Mullier

**Affiliations:** 1 Université catholique de Louvain, CHU UCL Namur, Namur Thrombosis and Hemostasis Center (NTHC), NARILIS, Haematology Laboratory, B-5530 Yvoir, Belgium; 2 Maastricht University Medical Centre and Cardiovascular Research Institute (CARIM), Department of Internal Medicine, Maastricht, The Netherlands

**Keywords:** Haemostasis assays, Pre-analytical phase, Coagulation assays, Recommendations, Dabigatran, Idarucizumab, Microparticles, Centrifugation

## Abstract

Ensuring quality has become a daily requirement in laboratories. In haemostasis, even more than in other disciplines of biology, quality is determined by a pre-analytical step that encompasses all procedures, starting with the formulation of the medical question, and includes patient preparation, sample collection, handling, transportation, processing, and storage until time of analysis. This step, based on a variety of manual activities, is the most vulnerable part of the total testing process and is a major component of the reliability and validity of results in haemostasis and constitutes the most important source of erroneous or un-interpretable results.

Pre-analytical errors may occur throughout the testing process and arise from unsuitable, inappropriate or wrongly handled procedures. Problems may arise during the collection of blood specimens such as misidentification of the sample, use of inadequate devices or needles, incorrect order of draw, prolonged tourniquet placing, unsuccessful attempts to locate the vein, incorrect use of additive tubes, collection of unsuitable samples for quality or quantity, inappropriate mixing of a sample, etc. Some factors can alter the result of a sample constituent after collection during transportation, preparation and storage.

Laboratory errors can often have serious adverse consequences. Lack of standardized procedures for sample collection accounts for most of the errors encountered within the total testing process. They can also have clinical consequences as well as a significant impact on patient care, especially those related to specialized tests as these are often considered as “diagnostic”. Controlling pre-analytical variables is critical since this has a direct influence on the quality of results and on their clinical reliability. The accurate standardization of the pre-analytical phase is of pivotal importance for achieving reliable results of coagulation tests and should reduce the side effects of the influence factors. This review is a summary of the most important recommendations regarding the importance of pre-analytical factors for coagulation testing and should be a tool to increase awareness about the importance of pre-analytical factors for coagulation testing.

## Background

The term “pre-analytical phase” describes all actions and aspects of the medical laboratory diagnostic procedure that occurs prior to the analytical phase [1].

This phase is part of the total laboratory procedure consisting of several stages and beginning with the physician requesting the performance of a laboratory investigation on a patient. It is the most vulnerable part of the total testing process, where most laboratory errors occur [2, 3]. It encompasses all procedures, starting with the formulation of the medical question, and includes patient preparation, sample collection, handling, transportation, processing, and storage until time of analysis.

Pre-analytical laboratory errors can arise throughout the pre-analytical phase, because this phase comprises a lot of manual activities and accounts for most of the errors encountered within the testing process as a whole. Unsuitable, inappropriate or wrongly handled procedures during collection and handling of specimens are very likely to lead to pre-analytical errors [3].

Problems may arise during the collection of blood specimens such as misidentification of the sample, use of inadequate devices or needles, incorrect order of draw, prolonged tourniquet placing, unsuccessful attempts to locate the vein, incorrect use of additive tubes, collection of unsuitable samples for quality (e.g. contaminated, haemolysed) or quantity (e.g. insufficient amount of blood or inappropriate blood-to-anticoagulant ratio), inappropriate mixing of a sample, etc. Some factors can alter the result of a sample constituent after collection during transportation, preparation and storage. The side effects of such factors of influence can be reduced by standardizing the pre-analytical process [4–6].

This review aimed at summarizing recommendations with regard to the pre-analytical phase and provides some guidance to reduce the effects of biological factors that can have a significant impact on patient care.

## Methods

A systematic review was conducted following the recommendations of the French Study Group on Haemostasis and Thrombosis (GFHT), the World Health Organization (WHO), the Clinical and Laboratory Standards Institute (CLSI) guidelines, the International Society on Thrombosis and Haemostasis (ISTH), the European Federation of Clinical Chemistry and Laboratory Medicine (EFLM) and the British Committee for Standard in Haematology (BCSH) about the pre-analytical phase and the pre-analytic variables which can have an impact on the quality of medical laboratory results and about the procedures for sample collection, processing, transportation, and storage in haemostasis testing.

The development of the present literature review was carried out using the PubMed database records including analysis of references from selected articles from 1991 to 2016 based on the following key-words: ‘pre-analytical phase’, ‘pre-analytic variables’, ‘pre-analytical quality’, ‘preoperative/pre-operative tests’, ‘order of draw’, ‘phlebotomy’, ‘screening tests’, ‘routine tests’, ‘screening testing’, ‘hemostasis/haemostasis’, ‘coagulation tests’, ‘bleeding history’, ‘preoperative bleeding questionnaire’, ‘preoperative evaluation’, ‘bleeding risk’, ‘haemorrhage/hemorrhage’, ‘surgery’, ‘von Willebrand disease’, ‘von Willebrand factor’, ‘inherited bleeding disorders’, ‘bleeding score’, ‘quality’, standardization’, ‘collection of blood’, ‘sample collection’, ‘tourniquet’, ‘samples’, ‘transportation’, ‘preparation’, ‘storage’, ‘recommendations’, ‘discard tubes’, ‘activated partial thromboplastin time, PTT, aPTT’, ‘prothrombin time, PT’, ‘international normalized ratio, INR’, ‘bleeding time’, ‘platelet count’, ‘platelet function testing’, ‘PFA-100’, ‘blood sampling’, ‘sample tubes’, ‘anticoagulant’, ‘order of filling the tubes’, ‘sampling process’, ‘processing of samples’, ‘transportation of samples’, ‘centrifugation’, ‘storage conditions’, ‘freezing’, ‘thawing’. Only articles in English or French were analysed. The database was searched for studies, clinical practice recommendations and literature reviews.

The findings of our search have been grouped into different subtopics. The first one is devoted to the clinical history, bleeding score and physical examination to demonstrate the importance of the patient’s history regarding various diseases such as thrombotic diseases in case of haemorrhagic diathesis or preoperative assessment. The following chapters concern the sample collection and sequence of drawing blood. The last subtopics concern the sampling process, transportation and storage conditions.

### The first step before considering laboratory testing; Clinical history and physical examination

Prior to any type of haemostatic testing a bleeding history is a prerequisite for the diagnosis of any bleeding disorder and should guide further laboratory investigations [7, 8].

If this is done diligently, it can be considered as one of the best screening tests of the risk of bleeding [7, 9, 10]. It allows the detection of pathology known to cause abnormal primary haemostasis (malnutrition, haematologic disease, liver failure, etc.). In case of acquired haemostatic disorders, family history can be seen as a family tree of the types of haemorrhage observed as well as their spontaneous or provoked features. As far as a person’s bleeding history is concerned, it is important to know the terms of appearance of haemorrhages, their frequency and their source, and whether bleeding is aggravated after taking aspirin and/or other medications like antiplatelet therapy, NSAIDs, etc. Regarding the surgical and obstetrical history, it is important to note the number of interventions that resulted in bleeding complications (new intervention due to complication, transfusion, admission to intensive care, etc.), but also the number of times that trauma was not associated with haemorrhagic complications. Antecedents of anemia, iron supplementation, hospitalization or blood transfusion can also guide the clinician [11, 12]. The patient’s blood group provides relevant information related to haemostatic activity (e.g. activity levels of VWF). Patients with blood group O are known to have lower levels of VWF. The presence of the *H-*antigen (O group) promotes the cleavage of VWF by ADAMTS13 metalloprotease, and, moreover, a higher hepatic clearance of VWF [13, 14].

In order to prevent disturbing pre-analytical influences, any interfering drugs should be administered after collecting a blood sample. A record of all the drugs that the subject took during the week prior to testing should be collected [15]. Treatment with desmopressin, in treating or preventing bleeding episodes in patients with von Willebrand disease, haemophilia A and platelet function defects, should be noted [16]. It is important to know if the patient is pregnant. Pregnancy is associated with increase in fibrinogen, factors VII, VIII, X, VWF, D-dimer concentration and with increase in levels of prothrombin fragments 1 + 2 and thrombin-antithrombin III complexes. There is a decrease in physiological anticoagulants manifested by acquired activated protein C (APC) resistance. Free protein S is decreased secondary to increased levels of its binding protein, the complement component C4b [17–19]. The overall fibrinolytic activity is impaired during pregnancy, but returns rapidly to normal following delivery. This is largely due to placental derived plasminogen activator inhibitor type 2 (PAI-2), which is present in substantial quantities during pregnancy [19]. Microparticles derived from maternal endothelial cells and platelets, and from placental trophoblasts may contribute to the procoagulant effect [19].

Hormonal contraceptives can be responsible for interferences in coagulation testing and may lead to increased concentrations of fibrinogen, prothrombin and factors VII, VIII and X, and reduction in coagulation inhibitors, such as antithrombin (AT), protein S and tissue factor pathway inhibitor (TFPI) [17, 20, 21]. Use of combined oral contraceptives is associated with a three-to six-fold increased risk of venous thrombosis. This increased risk depends on the estrogen dose as well as the progestogen type of oral contraceptives [22]. These changes tend to me more pronounced in women taking third generation combined oral contraceptives than on second generation [21]. Women using combined oral contraceptives with the highest risk of venous thrombosis (e.g. containing desogestrel, cyproterone acetate or drospirenone), have lower levels of PS and TFPI than women using the combined oral contraceptive with the lowest risk of venous thrombosis (i.e. contraceptives containing levonorgestrel) [22, 23].

Reduced concentrations of protein S can be caused by functional impairment of this protein by differences in modulation of its activity by other plasma proteins that change during the oral contraceptives use or by changes induced in the protein S molecule that impair its anticoagulant activity [24]. For platelet function assays, treatment with drugs known to reversibly inhibit platelet function (e.g. NSAIDs) should be stopped at least 3 days before sampling, and treatment with drugs known to irreversibly inhibit platelet function (e.g. aspirin, thienopyridines) should be stopped at least 10 days before sampling, if possible [8, 15]. As it oftentimes impossible to stop all medication before being sampled for coagulation studies, drug-induced effects on platelet function should be considered when interpreting results [15]. Treatment continuation is also sometimes recommended to assess the efficacy and safety of antithrombotic drugs [9].

The referring physician should also have done a proper physical examination in order to detect signs of coagulopathy. In case of primary haemostatic abnormalities, it is common to seek a cutaneo-mucous type of haemorrhage. Elements usually seen in haemorrhagic syndrome are bruising, purpura, or haemarthrosis. In case of testing for thrombophilia, signs of previous venous thrombosis including post-thrombotic syndrome (venous insufficiency, corona phlebectatica etc.) can be informative. Moreover, it is very important that the clinician and the laboratory exchange all relevant information as much as possible [25].

### Bleeding score

To collect the bleeding history, in case of haemorrhagic diathesis or preoperative assessment of bleeding risk, and to obtain the resulting bleeding score (BS), standardized and validated clinical tools are necessary [10].

*a) haemorrhagic diathesis:* In an attempt to standardize the diagnostic criteria of von Willebrand disease (VWD), the most prevalent congenital bleeding disorder which occurs with equal frequency among men and women and affecting up to 1% of the general population, a BS has been developed [26]. This one is useful for the identification of subjects requiring laboratory evaluation for VWD and for assessing the clinical severity of the disorder in patients with type 1 VWD [7, 27–29]. This BS, related to the number and the severity of bleeding symptoms, is based on a standardized questionnaire and used to evaluate haemorrhagic symptoms [7, 29]. The questionnaire has proven to be useful for diagnostic purposes, allowing the establishment of quantitative cut-offs discriminating healthy subjects and carriers of VWD [7]. The BS is proposed as a predictor of clinical outcomes of inherited VWD, and may also help to identify cases at higher risk of frequent and severe bleeds requiring more intensive prophylactic regimens [30].

Other BS have been developed for the large population of patients with atrial fibrillation (AF) that require oral anticoagulant therapy (e.g. HAS-BLED, ATRIA, ORBIT risk scores, etc.) [31].

The combination of the standardized bleeding questionnaire and an interpretation grid has been referred to as a Bleeding Assessment Tool (BAT) [7]. Although existing BS have limitations (e.g. dependence on clinician interpretation regarding patient recall, inability to distinguish among bleeding events occurring at different anatomical sites, etc.) their use is strongly encouraged, as BATs have proven validity in assessment of symptom severity and help identify patients needing further investigations [10, 32, 33].

To ensure reliability and reduce the time load, the questionnaire should be performed by a physician or other health professional experienced in assessing a (mild) bleeding disorder (MBD), particularly in patients who have abnormal clotting screening test results requiring invasive procedures or elective surgery [7, 34].

The ISTH/SSC Joint Working Group agreed to establish a single BAT to standardize the reporting of bleeding symptoms which would be useful for both paediatric and adult populations. More quantitative BATs have been proposed to improve the diagnostic criteria for MBD because most patients with an MBD do not show a definitive bleeding history, and are difficult to distinguish from normal subjects [7].

This revised BAT could be more generally applicable to the investigation of other inherited bleeding disorders with variable expressivity (e.g. mild platelet function disorders) to avoid the bias of subjective investigator evaluation of haemorrhagic symptoms and to reduce the need for expensive laboratory investigations [7, 8, 29]. Even if the ISTH-BAT has not been evaluated sufficiently in inherited platelet function disorders to allow a firm recommendation of its use, it could also be potentially useful for the diagnosis of platelet function disorders [32, 33].

*b) pre-operative assessment:* For preoperative investigations, some guidelines are available. Recently the French Society of Anaesthesia and Intensive Care issued recommendations for the prescription of routine preoperative testing before a surgical or non-surgical procedure, requiring any type of anaesthesia [9]. The aim of pre-anaesthetic screening, based on detailed patient interviews and on physical examination, is to identify patients with an increased risk of perioperative bleeding to minimise perioperative haemorrhagic complications.

Routinely haemostasis testing has very little therapeutic impact and lacks prognostic utility. Hence, haemostasis testing should not be systematically used in patients whose history and clinical examination results do not suggest any haemostatic disorders. Conversely, in patients with a positive history of haemorrhagic diathesis, haemostasis testing should be requested depending on the suspected disease [9].

These recommendations are in line with those issued in the United States and in the United Kingdom by the National Institute for Health and Clinical Excellence (NICE). Only the Italian SISET recommendations (Società Italiana per lo Studio dell’Emostasi et della Trombosi) suggest performing systematic haemostasis testing with aPTT, PT and platelet count prior to any intervention, even in patients with no bleeding diathesis history [9].

### Variables during sample collection

During the pre-analytical phase, in case of haemostasis testing for patients with a positive history of haemorrhagic diathesis, certain steps regarding the preparation of patients and the execution of sampling (specimen collection, transportation, sample preparation and storage) are of special importance. Providing patients with the appropriate instructions before blood sampling and proper training of those persons involved in sampling procedures can reduce or prevent negative influences on laboratory results and their misinterpretation [4, 35].

In order to minimize misinterpretation of laboratory results, blood samples should be collected from fasting subjects in the morning between 7 and 9 a.m. and from subjects who have refrained from smoking for at least 30 min [4, 8, 15, 36, 37]. Consumption of caffeine should be discouraged within the 2 h prior to sampling [4, 15]. Physical activity within the 2 h prior to sampling is not recommended. Blood samples should be collected after the subject has rested for a short period (at least 5 min) [15].

Diet and drinking are major factors influencing a number of analyses. Before blood sampling, the disturbing influences of food and drink should be excluded. Patients should not be studied after meals associated with a high fat content, so as to avoid the formation of chylomicrons in plasma, which will interfere with light transmission aggregometry [15, 37]. A light meal does not influence the laboratory coagulation tests. However, to completely metabolize the lipids intake, sampling after at least 8 h (better 12 h) fasting and reduced activity (bed rest) is recommended [4, 38]. It is also advisable not to eat dark chocolate [39].

Stress should be avoided under all circumstances (e.g. restless child), because stress increases acute phase proteins of which von Willebrand factor (VWF), factor VIII and fibrinogen [40] are most important (in particular in the workup for haemophilia or VWD). Smoking around the time of phlebotomy enhances platelets aggregability, and induces a procoagulant state in plasma [41, 42]. Studies have reported an increase in blood coagulability but impaired fibrinolysis in habitual smokers when compared with nonsmokers [42]. It seems that higher plasma levels of fibrinogen and viscosity are the main contributors to higher coagulability found in smokers, whereas the lower fibrinolytic potential is mainly attributed to an increase in PAI-1 activity and possibly also a decrease in tPA activity and lower plasminogen levels [41, 42]. Smokers may have impaired acute substance P-induced endothelial release of active tPA in vivo related to impaired endothelial function, which suggests a possible direct link among impaired endogenous fibrinolysis, endothelial dysfunction, and arterial atherothrombosis in smokers [42].

Caffeine enhances fibrinolytic potential as whole blood fibrinolysis time is shortened and PAI-1 levels are decreased, whereas tPA activity increases after consumption of coffee and such effects are blunted during caffeine abstinence [42]. About dark chocolate, platelet aggregation is modulated by a flavanol-independent mechanism that is likely due to theobromine which is containing by cocoa products [39].

Physical activity may cause an increase in leukocyte count and activation of coagulation (decrease of prothrombin time (PT) and fibrinolysis). It can also activate partial thromboplastin time (aPTT), cause an increase in D-dimer, tissue plasminogen activator (tPA) and plasminogen activator inhibitor (PAI), an exercise-induced adrenaline release on platelet aggregation and an increase in platelets [4, 15, 36]. Strenuous exercise also promotes the release of microparticles by platelets and triggers a transient procoagulant condition, which is mirrored by increased thrombin generation, platelet hyperreactivity, increased activity of clotting factors, compounded by an increased fibrinolytic activity [41, 43].

### Training in phlebotomy

Phlebotomy is the act of puncturing a vein for the purpose of withdrawing blood and is one of the most critical parts of the entire pre-analytical phase [44]. Training of phlebotomists is a pre-analytical challenge and requires continuous educational updates as equipment is changed in the healthcare institutions [45].

All staff members should be trained in phlebotomy, to prevent unnecessary risk of exposure to blood and to reduce adverse events for patients [46]. If the phlebotomist is a member of the laboratory staff, he/she will be aware of the impact of the quality of sampling on the quality of results. If he/she is external to the laboratory, the laboratory must provide all information necessary about good sampling practices [6, 36].

### Blood sampling

According to the recommendations of the EFLM, tube labelling must be done, before or after venipuncture, in the presence of the patient. The phlebotomist should label each drawn tube with the patient’s full name, patient’s date of birth, identification number [6, 38]. Of course, the use of prebarcode tubes may facilitate collecting this information. The sampling equipment must be sterile and non-pyrogenic. The expiry dates of the needle and the integrity of the sterile seal should be checked. The phlebotomy procedure provided by the CLSI requires wearing gloves, cleansing the venipuncture site and drying of skin before applying the tourniquet and selecting the venipuncture site and vein [47]. The venipuncture should be ideally collected directly from a peripheral vein (antecubital vein). If a tourniquet needs to be used, it should immediately be released when the first tube starts to fill [48]. The diameter of the needle should preferably be comprised between 19 and 22 gauge [34, 49].

It is important to take samples so as to reduce platelet activation in-vitro and to restrict the use of the tourniquet [15, 36, 49]. Extended tourniquet application might produce unnecessary venous stasis or in-vitro haemolysis, which could introduce spurious and clinically meaningful biases in the measurement of several haematologic parameters [47]. Hence, the tourniquet should be placed tightly but less than one minute in order to prevent haemoconcentration, increased fibrinogen and factors VII, VIII, XII as well as activation of endothelial cells and therefore fibrinolysis [47, 48]. Some special coagulation assays, such as those that measure thrombin generation markers (e.g., thrombin antithrombin complex (TAT) and prothrombin fragment 1 + 2), should be drawn without the use of a tourniquet because that may lead to spurious elevation of these markers, particularly if the tourniquet is left in place for more than one minute [5]. A quality laboratory manager should verify the length of tourniquet application and forearm clenching in order to eliminate this source of laboratory error and safeguard quality throughout the total testing process [48].

Venipuncture may be necessary, on occasion, to obtain blood from an existing vascular access device such as an intravenous (IV) line or a central line [5]. If an IV line is present, the sample site selected is distant from the line.

Butterfly devices and 23 gauge needles can be used on patients with difficult veins (geriatrics, oncology and paediatrics), provided that the tubing is short (length <6 cm and air volume <150 μL) [36, 45].

However, owing to costs and the risk of obtaining unsuitable samples, the use of butterfly needles and IV catheters has generally been discouraged, because haemolysis, activation of the contact system, initiated by activation of factor XII, can occur when blood comes into contact with the surface of a biomaterial [2]. Blood samples should be obtained in a relatively atraumatic fashion, and during collection the blood should flow freely into the collection container [4]. In case of difficult sampling, at least this information should be recorded for adequate interpretation of haemostasis assays [50]. Blood sampling should be performed without generating foam or bubbles in the sample and with as little shear stress as possible [4].

The passage of blood through butterfly tubing and IV catheters might cause increased haemostatic alterations in comparison to blood collection using a conventional straight needle, directly into the tube [2].

The direct transfer of blood specimens from syringes to blood collection tubes by piercing of the rubber stopper of the tube is a practice that should be avoided. This may cause haemolysis when cells under pressure from the plunger collide with the tube wall [4].

The investigation of platelets is highly vulnerable to a broad series of pre-analytical variables [8]. In particular, the vacuum aspiration of blood into primary collection tubes could have an influence on platelet function testing. A syringe system permitting slow manual drawing of blood may be superior [4]. For the Platelet Function Analyser (PFA-100), which replaces the bleeding time in many laboratories, the lower shear stress generated by manual aspiration of blood into the primary collection tube would prevent spurious hyper-activation of platelets, thus, preserving the integrity of their function for subsequent testing on PFA-100 [8, 27, 51].

Optimally, blood sampling is done in the laboratory performing the analyses, which allows for verifying many of the above mentioned steps including when needed getting additional data on medical history, medication, fasting state etcetera. Patient identification and tube labelling are the most critical steps during phlebotomy, providing an essential safety barrier to prevent patient identity mix-up.

Labelling blood tubes in the absence of the patient is a potentially life threatening error. According to CLSI, patient identification is the responsibility of the phlebotomist to ensure that blood is drawn from the individual designated on the request form [6] (Table 1).

**Table 1:** Summary of key pre-analytical recommendations about blood sampling

Blood sampling	Use siliconized glass or plastic (polypropylene) tubes.
	Blood samples should be drawn into 105–109 mmol/L sodium citrate, buffered anticoagulant.
	The pH of the anticoagulated plasma should be comprised between 7.3 and 7.45.
	Perform blood collection from fasting subjects in the morning (between 7 and 9 a.m.).
	The patient should be relaxed. Stress should be avoided.
	Label each drawn tube with the patient’s full name, patient’s date of birth, identification number.
	Collect venipuncture directly from a peripheral vein (antecubital vein).
	The diameter of the needle should preferably be comprised between 19 and 22 gauge.
	Release the tourniquet immediately when the first tube starts to fill (<1 min).
	The order of drawing blood during phlebotomy should be blood culture/sterile tubes, then coagulation tubes, then plain tubes/gel tubes, then tubes containing additives.
	Draw a discard tube when citrated plasma is obtained using butterfly systems or other IV catheter devices.
	Discard tube may be considered to ensure correct filling of sample tubes for coagulation tests.
	Ensure correct filling of tubes (>90% filling).
	Respect the required ratio of sodium citrate to whole blood (1:9).

### Sample tubes and anticoagulant

Blood samples for coagulation analyses should be drawn into siliconized glass tubes, or plastic (polypropylene) tubes [35, 49]. Vacuum tubes must be sealed and CE marked [15, 36]. Care must be taken to follow manufacturer’s expiry dates. Sample tubes for haemostasis analyses generally contain an anticoagulant. The CLSI guidelines on the collection of blood specimens for coagulation testing recommend the use of sodium citrate in 105–109 mmol/L (3.2%) tubes. The use of mmol/L should be preferred to %. The pH of the anticoagulated plasma should be comprised between 7.3 and 7.45 [35, 36].

The vast majority of the samples should be collected into trisodium citrate, buffered anticoagulant to help keep the pH stable during processing and testing [15, 36]. The anticoagulant effect of sodium citrate is attributed to its ability to bind calcium, making the calcium unavailable to promote clot formation [4]. However there may also be higher citrate concentration (i.e., 3.8% or 129 mmol/L) leading to greater calcium binding and longer clotting times. Specimens collected in 3.8% buffered sodium citrate may prolong the PT and aPTT and underestimate fibrinogen if the normal range is based on 3.2% citrated samples [4, 47]. Due to the variation in clotting times and sodium citrate concentration, the consensus recommendation suggests that laboratories should standardize to one citrate concentration and develop appropriate reference intervals [4, 5, 35, 36, 47].

For some analyses, especially platelet function assays, buffered citrate solution or other anticoagulants are used. Citrate, theophylline, adenosine and dipyridamole (CTAD) is a cocktail of additives that prevent in-vitro platelet activation recommended for measurement of platelet-activation markers such as β-thromboglobulin or platelet factor 4 (PF4). These tubes allow for more reliable measurement of unfractionated heparin (UFH) and are useful for the study of membrane glycoproteins platelet by flow cytometry. They should be kept away from light [36]. Sampling these tubes allows a delay in analysis until 4 h before centrifugation [52].

It is easy to ensure that the anticoagulant is consistent when working on the primary tube; vigilance is therefore required in case of analysis performed on an aliquot [53].

### Sequence of drawing blood

A standardized sequence of blood sampling must be respected in order to avoid carry-over between tubes [3]. National and international (WHO, CLSI) guidelines recommended that the order of draw of blood during phlebotomy should be blood culture/sterile tubes, then coagulation tubes, then plain tubes/gel tubes, then tubes containing additives [3–5]. According to the CLSI, a first discard tube (or a non-additive tube) is unnecessary for routine coagulation assays [47]. A discard tube (or a citrate tube if the sampling is difficult) must be drawn when citrated plasma is obtained using butterfly systems or other IV catheter devices because the air volume contained in the tubing partially fills the vacuum tube, leading to insufficient filling of the tube with citrate. A discard tube is also recommended when samples are subject to platelet function analysis and for thrombin generation measurements [2, 4, 8, 47].

Published studies have demonstrated that for routine coagulation testing, the use of a discard tube is not necessary because there was no significant difference in the aPTT and PT results between the first and second tubes drawn [2, 4, 5, 36, 44, 54]. Some studies evaluated the need for discard tubes in a variety of others coagulation tests (e.g. fibrinogen, D-dimer, factors II, V, VII, VIII, IX, X, XI, proteins C and S and AT, …) and suggested that discard tubes are not necessary when drawing samples for specialized coagulation testing [2, 5, 54, 55]. There are no data to support the need for a discard tube for specialized haemostasis assays [5]. However, due to lack of sufficient evidence, the practice of drawing a discard tube should still be recommended [5].

Contamination can occur through contact from micro drops from caps with a compress, or from contact with the interior of the tube with the syringe and from the anticoagulant of the first tube to the second tube [4]. Contamination of coagulation assay tubes is possible if coagulation tubes are drawn following an additive tube like certain serum collection tubes containing clot activators [36]. In case of contamination, the results will be erroneous and a re-collection will be necessary, leading to a delay in subsequent medical decisions.

For platelet function assays and coagulation factors, we suggest numbering each tube according to the sample collection order. If a defect is observed on one tube, it could be useful to check this number and verify on another tube.

### Sampling process

#### Filling the tubes

Sodium citrated tubes must be filled up to 90% of the nominal volume or to the mark noted on the tube if provided [41, 47]. The required ratio of sodium citrate to whole blood is 1:9 [35, 49].

Under-filling of tubes is another important source of error and severely affects laboratory results. An insufficient volume for testing greatly modifies the fixed blood-to-anticoagulant ratio. Under-filling increases the dilution of the sample due to the volume of liquid anticoagulant, and may increase the clotting time due to the excess calcium-binding citrate present [4, 5, 47].

It has been reported that when tubes are drawn at less than 89% of total fill, a clinically significant bias exists in test results for aPTT, less than 78% for fibrinogen, and less than 67% for coagulation factor VIII, whereas PT and activated protein C resistance remain relatively reliable even in tubes drawn at 67% of the nominal volume [41]. Similar results were obtained in further studies [45, 56]. Instead of rejecting a sample tube, it may be useful to note the sample volume and to adapt the result depending on the additional dilutional (e.g. for factor assays, fibrinogen). In case of high haematocrit, the citrate volume may be adjusted because this may also impact the citrate-calcium ratio. It may be necessary to remove part of the citrate solution from the sampling tube prior to drawing blood [35, 36, 44] (Table 2).

**Table 2:** Summary of key pre-analytical recommendations about sample processing

Sample processing	Blood should be adequately and promptly mixed by 3 to 6 complete end-over-end inversions of the tubes in order to ensure complete distribution of anticoagulant.
	Avoid vigorous shaking, vortexing or agitation of blood samples.
	Adjust the citrate volume in case of high haematocrit (remove part of the citrate solution from the sampling tube prior to drawing blood).
	Check tubes for presence of clots, precipitates or haemolysis.

#### Mixing the samples

Following collection, blood should be adequately and promptly mixed by three to six complete end-over-end inversions of the tubes in order to ensure complete distribution of anticoagulant [8, 36, 47, 49, 57].

Mixing samples is an important way to prevent in-vitro clot formation. Sometimes samples are inappropriately mixed or left unmixed for a long time, thereby avoiding full contact of the blood with the anticoagulant, which determines partial clotting.

Vigorous shaking, vortexing or agitation of blood samples should be avoided in order to prevent inducing haemolysis or spurious platelet and factor activation that may result in shortened clotting times or false elevation of clotting factor activity in specimen tests (e.g. factor VII) [4, 8, 48, 58].

#### Examination of samples

When obtaining plasma for coagulation testing and prior to processing, samples must be examined for the presence of a clot, precipitates and signs of haemolysis.

In-vitro clots may develop in samples where the blood is slow to fill the collection container, where there is prolonged use a tourniquet, or when considerable manipulation of the vein by the needle has occurred. These situations must be avoided.

Several causes may induce in-vitro haemolysis, such as slow or difficult specimen collection; prolonged tourniquet placing; use of incorrect devices (e.g., butterfly needles or IV catheters) or needles (e.g., small gauge needles); unsuccessful attempts to locate the vein; inappropriate mixing of the sample; inappropriate centrifugation speed (e.g., > 1500g); inappropriate transportation procedures (pneumatic tube systems, duration, temperature and humidity), … [2, 4, 5].

Haemolysis can affect some tests of haemostasis, either because of the presence of thromboplastic substances or interference of haemoglobin pigment with photo-optical systems [2, 49].

Lysis of the red cell membranes induces the release of red cells contents (many intracellular enzymes, ADP …) into plasma and may lead to activation of the plasma sample altering coagulation parameters and activation of other bloodlines (leucocytes, platelets).

Haemolysed samples may lead to early flow obstruction in the PFA, presumably due to platelet activation, fragmentation of platelet and red blood cells and the presence of micro-thrombi [4]. Haemolysis may lead to statistically significant increases in PT and D-dimer. The aPTT can be falsely prolonged or shortened and AT and fibrinogen decreased by in vitro haemolysis [5, 49, 59, 60].

Plasmas that are lipemic and icteric may also affect analytic results by interfering with optical absorbance or impeding light transmittance [49]. Mechanical and/or electromechanical methods for clot detection should be utilized when possible for these plasma samples [5]. Because the presence of lipid particles can still bias the measurement for biologic interference, the CLSI currently recommends the removal of excess triglycerides by ultracentrifugation [61]. This kind of equipment being unavailable in most routine laboratories due to high costs and incompatibility with daily practice and because ultracentrifugation may cause precipitation of the large molecular proteins such as fibrinogen or factor VIII/VWF complex, an alternative approach entails high speed microcentrifugation (e.g., double centrifugation at >20,000 g for 15 min) or lipid extraction by means of organic solvents or lipid-clearing agents [45, 61].

### Transportation of samples

Samples should be transported non-refrigerated at ambient temperature (15–25 °C) in as short a time as possible [8, 49, 57].

Before transport, samples should be tested regarding identification, safety conditions and stability. Errors in either sample identification, sample preparation before or after transport may have an adverse effect on patients if they are not detected on time [4, 6].

When possible, samples should be drawn directly in a laboratory. Immediately after drawing, whole blood should remain capped for transport both for safety reasons and to minimize loss of CO2, which causes pH to increase, leading to prolongation of PT and/or aPTT [44]. Tubes should be stored at ambient temperature until centrifugation [4, 5, 36]. Temperature control is recommended in rooms where samples are kept for analysis [2]. If tubes need to be transported this should be done with care in order to avoid unnecessary agitation. During transportation, samples should be transferred vertically in the shortest possible time [36].

Delays between sample collection and analysis can cause in-vitro degradation of coagulation proteins [2].

Ideally, samples for coagulation assays should be performed in the laboratory that performs the assays. If not, specimens should be shipped from peripheral collection facilities to the core laboratory utilizing current CLSI guidelines (non-refrigerated at ambient temperature in as short a time as possible, preferably within the first hour after collection) [5, 57]. Blood samples for coagulation analyses should not be shaken. Any sample that has been dropped should be discarded. For the transport, a box maintaining blood tubes in a steady vertical position should be used. Transporting blood tubes in a vertical rather than a horizontal position limits the extent of in vitro microparticles (MP) generation [62, 63].

The temperature during transport is of special relevance. Extreme temperatures should be avoided in order to maintain sample integrity [5]. Temperatures higher than room temperature can lead to degradation of factor V and factor VIII [64].

The use of pneumatic tube systems (PTS) for transport of samples is problematic and could have a significant influence on platelet function testing [8, 45]. Rapid acceleration and deceleration may induce excessive vibration, denature proteins and result in haemolysis, platelet activation, and other effects [4, 35, 63]. Clinical decisions regarding platelet function and aspirin responsiveness should not be based on blood specimens transported by a PTS [65–68] (Table 3).

**Table 3:** Summary of key pre-analytical recommendations about transportation of samples

Transportation of samples	Before transport, test samples regarding identification, safety conditions and stability.
	Transport samples at ambient temperature (15–25 °C) in as short a time as possible.
	Draw samples directly in a laboratory.
	Immediately after drawing, whole blood should remain capped for transport.
	Temperature control is recommended in rooms where samples are kept for analysis.
	Transfer samples vertically.
	Do not use pneumatic tube systems (PTS) for transport of samples used for platelet function analysis.

### Specimen rejection

Each laboratory should have guidelines for rejection of samples; some criteria are obligatory: inappropriate collection tubes and additives, outdated tubes, error in patient identification or lack of identification, insufficient volume, haemolysed specimens, identification of a clot, inadequate volume, haematocrit >50% or <30% for the PFA, platelet count <100,000/μL for PFA and platelet aggregation … All samples deemed unacceptable due to pre-analytic handling and unfulfilled transport requirements should be rejected [4–6, 8].

Haemolysed blood is the most common reason for rejecting specimens in the laboratory and, therefore, in-depth knowledge on how to properly access the vein with the correct blood collection equipment to avoid haemolysis in the collected specimens is a must for the phlebotomist [45].

In case of reception of an aliquot without information about the anticoagulant used, this one must be rejected because serum and all types of plasma have virtually identical visual appearance. Because samples that are rejected must be recollected, which gives potential delay in patient management, phlebotomists must be fully aware of the common reasons why specimens are rejected (Table 4).

**Table 4:** Summary of key pre-analytical recommendations about specimen rejection

Specimen rejection	All samples deemed unacceptable due to pre-analytic handling and unfulfilled transport requirements should be rejected.
	Inappropriate collection tubes and additives.
	Outdated tubes.
	Error in patient identification or lack of identification.
	Insufficient volume (depending on the assay). An alternative may be to adapt the result depending on the additional dilutional (e.g. for factor assays, fibrinogen).
	Haemolysed specimens (depending on the assay).
	Identification of a clot.

### Centrifugation

Whenever a delay in transport is expected, it might be advisable to perform local centrifugation and separation. Plasma is generally prepared by centrifugation of a whole blood sample. A temperature-controlled centrifuge is required for processing routine coagulation assays. Centrifugation should take place at room temperature (15–25 °C) [36]. The effect of centrifugation temperature on MP determination is still unknown [37].

It is recommended to use a centrifuge that has a rotor with swing-out buckets to facilitate the separation of plasma from the cellular components and to minimize re-mixing of plasma and red cells [5, 36, 44]. It is recommended to centrifuge the primary tube for coagulation testing at 1500g for no less than 15 min with a centrifuge brake set off [8, 36, 47]. The centrifuge should be validated before use, every 6 months or after modifications, in order to assure that platelet-poor plasma (PPP) is achieved [47].

Use of the rotor brake may lead to higher residual microparticles and platelet counts, which also has an effect on PT and fibrinogen concentration [69]. Therefore, it seems feasible to centrifuge samples for coagulation analyses with the rotor brake set to off [4, 62]. However, according to our personal experience, this should be validated in appropriate studies. Our experience shows it is important to check the absence of vibration (during acceleration/deceleration processes) due to lack of centrifuge maintenance.

Using relative centrifugal forces greater than 1500g is not recommended as this may induce platelet activation, haemolysis or other unwanted effects [4, 5, 47]. However, in case of emergency, for general haemostasis parameters performed on fresh plasma, higher centrifugation force (greater than 1500g) and shorter time (less than 10 min) can be used [36, 47].

Some studies have evaluated the impact of high acceleration centrifugation conditions on routine coagulation testing (including especially PT, aPTT and fibrinogen; no data were found for the thrombin time) and concluded that rapid centrifugation does not modify results and contributes, by decreasing duration of the pre-analytical variable, to reduce the turnaround time for these tests [70–76]. Note that in the REVERSE-AD study conducted to evaluate the efficacy and safety of Idarucizumab, the reversal agent for dabigatran [77], the centrifugation (i.e. 3 min at 1000g) used before performing dilute thrombin time and ecarin clotting time, was not validated [78]. Both tests were used as primary endpoints and they should thus be interpreted cautiously.

*a) For analysis in platelet-poor plasma* For analysis in PPP, which is the standard material required for PT, aPTT, fibrinogen, single coagulation factor assays, functional protein S and C assays, activated protein C resistance, lupus anticoagulant (LA) assays, antiphospholipid antibody testing, thrombin generation, microparticles measurements, homocysteine, VWF assays, tPA, PAI, plasminogen and antiplasmin, or monitoring of unfractioned heparin therapy, and many other tests, sample tubes require a second centrifugation and should be centrifuged in capped tubes within 4 h after drawing blood [2, 36, 37, 79]. For thrombin generation measurements, whole blood is best centrifuged immediately, to prevent activation or degradation of coagulation proteins. In order to eliminate platelet debris and microparticles from plasma, which may contribute to the variability in thrombin generation results, a second centrifugation step at 10,000g is recommended [2].

Double centrifugation significantly reduces the residual amount of platelets in a sample and can be performed to produce PPP such that the post centrifugation plasma platelet count is less than or equal to 10x10^9^/L. Residual platelets in plasma have been known to affect phospholipid-dependent coagulation tests through the exposure from platelet membranes of anionic phospholipids that quench LA activity. This effect leads to shorter coagulation time and is particularly evident, especially in test plasmas that undergo freezing and thawing before analysis. Adequate plasma preparation for LA testing is an essential prerequisite for reliable diagnosis [80].

The generation of PPP should be prepared for all coagulation parameters if there is a possibility that measurement is not performed immediately after centrifugation, or if samples are going to be frozen [5, 63].

Following initial centrifugation, the plasma is carefully transferred to a nonactivating plastic centrifuge tube using an automatic pipette, and then centrifuged again for about 15 min. When pipetting, 1 cm of plasma must remain above the buffy layer.

It is important to use an automatic pipette because it permits slow and linear suction, unlike plastic pipettes that pose a risk of getting a high rate of residual platelets. The plasma is aliquoted to a secondary tube, taking care not to include the residual platelets that may have precipitated at the bottom of the centrifuge tube. It has been demonstrated that a high platelet count (>200 × 10^9^/L) does not affect results of PT, D-dimer, fibrinogen and aPTT assays, when samples are tested fresh and analyzed immediately after centrifugation [36, 79]. These samples should not be stored for later analyses [47].

*b) For analysis in platelet-rich plasma* The preparation of platelet-rich plasma (PRP) for platelet function analysis requires that centrifugation is performed at 200–250 g for 10 min without application of a rotor brake [8, 15]. These centrifugal forces appear to be the best condition for preparing PRP for light transmission aggregometry (LTA) studies, both in terms of the degree of contamination of PRP by other blood cells and of platelet reactivity [15]. The recommendation to avoid using a rotor brake is based on expert opinion and should be demonstrated in appropriate studies.

Centrifugation with less than 150 g does not yield a sufficient volume of PRP. The PRP should then be transferred to a capped polypropylene tube and a platelet count performed.

For LTA, the platelet count of PRP samples should not be adjusted to a standardized value by addition of autologous PPP, unless the platelet count is >600x10^9^/L [8, 15]. However, the results of LTA studies could be inaccurate when the platelet count in the PRP samples is lower than 150x10^9^/L [8, 15].

All assays using whole blood or PRP need to be performed within a maximum of 4 h after blood sampling [4, 15].

If the pre-analytical phase of the laboratory is supported by an automated system, vigilance must be in order to test and validate the centrifugation conditions meeting the requirements of haemostasis but also the requirements of chemistry and immunology analysis [36] (Table 5).

**Table 5:** Summary of key pre-analytical recommendations about centrifugation

Centrifugation	Use a temperature-controlled centrifuge for processing routine coagulation assays.
	Validate the centrifuge before use, every 6 months or after modifications, in order to assure that platelet-poor plasma (PPP) is achieved.
	Check the absence of vibration (during acceleration/deceleration processes) due to lack of centrifuge maintenance.
	Centrifuge the primary tube for coagulation testing at 1500g, 15 min
	In case of emergency, for PT, APTT and fibrinogen performed on fresh plasma, higher centrifugation force (greater than 1500g) and shorter time (less than 10 min) can be used. The preparation of PPP require a double centrifugation to obtain a residual platelet count lower than 10x10^9^/L.
	Following initial centrifugation, transfer carefully the plasma to a nonactivating plastic centrifuge tube using an automatic pipette, and then centrifuged again for about 15 min.
	The preparation of platelet-rich plasma (PRP) for platelet function analysis requires a centrifugation performed at 200–250 g for 10 min without application of a rotor brake.

### Storage conditions

*a) conditions for the interval from sampling to analysis:* For routine coagulation testing like the PT and the aPTT, storage of uncentrifuged samples at room temperature up to 6 h may yield acceptable results. However, a shorter delay is desirable. Whole blood assays should be performed within 4 h after blood sampling and centrifugation should ideally be taken within 1 h [4, 41, 47, 49, 79, 81]. During storage, samples should remain capped [5]. Extremes of temperature (e.g., both refrigerated or high) should be avoided.

Cold storage of citrated whole blood prior to centrifugation, by placing samples either in an ice bath or in refrigerated (2–8 °C) storage, is no longer recommended. Improper storage of whole blood at cold temperature may cause VWF and factor VIII values to fall below normal reference threshold levels, which may potentially lead to a false suspicion of haemophilia A or VWD due to inappropriate pre-analytical handling of blood [4, 5, 25, 44, 82].

Once the blood sample has been centrifuged, plasma can remain on the cells in a capped primary tube until testing, or it can be aliquoted and stored in a secondary tube. When aliquoting the plasma, care must be taken so that the buffy coat (the layer of cells between the red cells and plasma) is not disturbed or that this cellular component is not introduced back into the plasma. Aliquoted plasma is stable for 4 h when refrigerated, except for plasma for PT-INR and PT-sec, which should not be refrigerated [2, 5, 47]. This is due to the potential for cold activation of a sample. This may strongly influence some of the coagulation assays and may lead to platelet activation, activation of factor VII, which in turn can give shorter clotting times and hence lower PT-INR and PT-sec results.

Delays in transport may affect in particular the labile factors (FV, FVIII), leading to prolonged clotting times and in vitro loss of factor activity [2, 83].

The time frame for analysis depends upon the stability of the analysis, which depends on storage temperatures and conditions. As far as the stability of citrated whole blood, plasma on cells and aliquoted plasma is concerned, more studies are needed, because there are large differences between the conclusions of different studies and partially missing studies.

*b) short term plasma storage:* PPP should be stored at room temperature (20–25 °C) or at −80 °C until analysis. External influences (like temperature, light …) may be of major impact (Table 6).

**Table 6:** Summary of key pre-analytical recommendations about storage conditions

Storage conditions	Store samples at room temperature (15–25 °C) until analysis.
	Perform whole blood assays <4 h after blood sampling and centrifugation <1 h.
	Extremes of temperature (e.g. both refrigerated or high) should be avoided.
	Store PPP at room temperature (15–25 °C) or at −80 °C until analysis.
	If the whole blood sample is centrifuged within 1 h of collection, the plasma can be left on top of the cells at room temperature up to 4 h prior to testing.
	Time from sampling to analysis depends on analyte: Samples for PT/INR have longer stability (24 h) at room temperature. Samples for aPTT should be performed using fresh plasma <4 h (<1 h in patients treated with unfractionated heparin). For platelet function assays, samples should rest at room temperature for at least 15 min before analysis. Testing should be completed <3–4 h of collection. For factors V and VIII analyses should be performed <3 h. Fibrinogen, protein C and antithrombin activity appear to remain relatively constant when stored at room temperature for up to 7 days. Protein S activity is unstable, with a statistically significant loss of activity at 8 h. VWF appears to be stable at room temperature for 48 h.

Samples for PT/INR have longer stability (24 h) at room temperature. Samples can be stored as whole blood or stored following centrifugation [81].

Samples for aPTT testing should be stored at room temperature and be performed using fresh plasma within 4 h. The limited stability is largely due to time-dependent generation or loss of labile factors, particulary factor VIII and possibly factor V [83]. aPTT from patients receiving unfractionated heparin (UFH) therapy has shorter stability. For these samples, due to the variable heparin neutralization by platelet factor 4 (PF4), the delay before centrifugation should not exceed 1 h for a sample collected in citrate and 4 h in CTAD. If the whole blood sample is centrifuged within 1 h of collection, the plasma can be left on top of the cells at room temperature up to 4 h prior to testing [83]. This applies also to fibrinolysis parameters [35, 47, 52, 81, 83].

PT, aPTT and factor VIII tests from frozen samples cannot be performed [4]. Freezing has an inconstant and unpredictable effect on the results and may cause significant elevations of aPTT, but also PT [4, 84–86]. Poorly handled frozen material may also causes shortening of aPTT or PT [5]. If double centrifugation is not done when preparing a sample to be frozen this can lead to a lysis of residual platelets upon freezing of plasma sample and lead to shortened APTT results in heparinized patients [80].

Freezing leads to a marked decrease especially in FVIII activity [4, 87]. In case of a factor defect observed on a frozen sample, it is suggested to repeat the analysis on a fresh sample.

For platelet function assays, samples should rest at room temperature for at least 15 min before analysis. Testing should be completed within 3 to 4 h of collection [4, 5, 8, 15].

Stability of plasma samples for special coagulation assays (except FVIII, anti-FXa for UFH) is largely unknown. The shortest stability is observed for factors V and VIII. Analyses should be performed within 3 h.

For unknown reason, protein S is labile. It has been demonstrated that protein S activity is unstable, with a statistically significant loss of activity demonstrated at 8 h while fibrinogen, protein C and antithrombin activity appear to remain relatively constant when stored at room temperature for up to 7 days [83]. VWF appears to be stable at room temperature for 48 h [4].

Stability of the vitamin K-dependant factors has been reported to be 24 h at room temperature [4, 5, 83, 88]. For thrombin generation, direct plasma preparation is preferred to whole blood storage. When immediate analysis is not possible, plasma is most stable when incubated at room temperature, instead of 4 °C or 37 °C, most likely because of more activation and degradation of coagulation proteins at these temperatures [2].

For samples coming from external laboratories, it is important to check prior to analysis whether the aliquot external samples (fresh or frozen) really consist of citrated plasma. For specialized coagulation testing, most samples are sent as frozen samples, whilst samples for routine coagulation are more often sent as citrated blood (primary tube). Ideally, the primary tube should be received together with the aliquot tube [39]. It is not suggested to recentrifuge the aliquots coming from external laboratories.

*c) long term plasma storage:* Samples that cannot be tested within 4 h should be centrifuged and the plasma aliquot frozen [79] (Table 7).

**Table 7:** Summary of key pre-analytical recommendations about freezing and thawing

Freezing and thawing	Do not perform PT, aPTT and factor VIII tests from frozen samples.
	Centrifuge samples that cannot be tested within 4 h and frozen the plasma aliquot.
	Use rapid freezing technique (liquid nitrogen).
	Store samples at −70 °C (or below) rather than −20 °C.
	Plasma samples frozen at minus 20 °C remain stable for 2 weeks.
	Plasma frozen at minus 80 °C remains stable for 6 – 18 months dependent on the parameter.
	Do not re-freeze samples (but prepare a sufficient number of aliquots).
	Thaw samples rapidly at 37 °C (to prevent denaturing fibrinogen) at least 5 min in a water bath at 37 °C and not at room temperature, on a bench or in a microwave oven. Test immediately.
	After thawing, mix the sample gently to resuspend any cryoprecipitate. Do not vortex or shake.
	Do not re-frozen samples.

For analyses that can be performed on frozen plasma, freezing should be fast (using rapid freezing technique like liquid nitrogen), and samples should be preferably stored at −70 °C (or below) rather than −20 °C. Plasma samples frozen at minus 20 °C remain stable for 2 weeks for most coagulation parameters [36, 49]. However, it is imperative that a frost-free freezer is not used [36, 44, 49, 83]. Plasma frozen at minus 80 °C remains stable for 6 – 18 months dependent on the parameter [47, 63, 64].

Prior to testing, frozen plasmas should be thawed rapidly at 37 °C (to prevent denaturing fibrinogen) and tested immediately. This usually takes at least 3–5 min for a 1–2 mL sample. The sample must be mixed gently to resuspend any cryoprecipitate [49].

The samples have to be thawed at least 5 min in a water bath at 37 °C and not at room temperature, on a bench or in a microwave oven. After thawing they should be gently stirred [2, 36]. After thawing, and for thrombin generation measurements, plasma is best analysed immediately [2]. Freeze-thawing may produce phospholipid rich membrane microvesicles from platelet damage which may then mask the presence of a lupus anti-coagulant [89].

If a pathological parameter is obtained on a frozen sample, this parameter is suggested to be re-tested on fresh plasma. Samples may not be re-frozen. Several aliquots (suggested volume of 500–1200 μL) should be prepared as a backup. Frozen aliquots should be transported on dry ice [36].

## Conclusion

Continuous monitoring and management of pre-analytical errors is crucial in order to improve the quality of the pre-analytical phase, which is essential for patient care. Standardization efforts are essential to control and prevent errors and to ensure the quality of exploration in haemostasis. They are also necessary for all clinical laboratories accredited by International Organization for Standardization (ISO) document 15189. The accurate standardization of the pre-analytical phase is of pivotal importance to achieve reliable results of coagulation tests.

Owing to the development of large laboratory networks and of decentralized phlebotomy services and analytical laboratories, standardized and unequivocal procedures and protocols are essential for sample collection, including patient preparation, specimen acquisition, handling and storage. These procedures are intended to prevent these problems and to protect against complications and patient mismanagement that could otherwise arise when specimens are not collected properly in order to achieve accurate and reliable coagulation measurements.

The effects of pre-analytical variables on the reliability and consistency of screening tests is often forgotten due to a lack of understanding and awareness. This can be improved by educating healthcare professionals who are involved in drawing blood for testing.
